# zDHHC21 loss of function activates lung fibroblasts by regulating PTEN palmitoylation

**DOI:** 10.1093/ajrcmb/aanag097

**Published:** 2026-05-17

**Authors:** Bieerkehazhi Shayahati, Aaron K McDowell-Sanchez, Askar M Akimzhanov, Konstantin Tsoyi

**Affiliations:** Department of Biochemistry and Molecular Biology, University of Texas Health Science Center Houston, McGovern Medical School, Houston, TX, United States; Division of Pulmonary, Critical Care and Sleep Medicine, Department of Medicine, University of Maryland School of Medicine, Baltimore, MD, United States; Section of Pulmonary, Critical Care, and Sleep Medicine, Department of Medicine, Baylor College of Medicine, Houston, TX, United States; Department of Biochemistry and Molecular Biology, University of Texas Health Science Center Houston, McGovern Medical School, Houston, TX, United States; Division of Pulmonary, Critical Care and Sleep Medicine, Department of Medicine, University of Maryland School of Medicine, Baltimore, MD, United States; Section of Pulmonary, Critical Care, and Sleep Medicine, Department of Medicine, Baylor College of Medicine, Houston, TX, United States

To the Editor:

Idiopathic pulmonary fibrosis (IPF) is featured by excessive collagen-rich extracellular matrix (ECM) proteins deposition and lung remodeling which may lead to its failure.^1^ Fibroblasts are known to play one of the major roles in the progression of pulmonary fibrosis by overexpressing ECM proteins, myofibroblast transformation, and resistance to apoptosis in the profibrotic milieu.^1^ Thus, the attenuation of fibroblasts’ profibrotic activities is one of the strategies for therapeutic intervention to treat IPF.

Protein palmitoylation, also known as S-acylation, is a reversible posttranslational modification of cysteine residues with long-chain fatty acids.^2^ In mammalian cells, protein palmitoylation is facilitated by Zinc finger Asp-His-His-Cys (DHHC)-type acyltransferases (zDHHCs), which catalyze the addition of fatty acid chains to proteins.^3^ Several profibrotic signaling proteins, such as STAT3, ERK1/2, Akt, and mTOR, have been previously identified as palmitoylated, suggesting that both zDHHCs can play critical roles in profibrotic signaling pathways.^4-6^ This idea is supported by recent studies assessing the functional role of zDHHCs in experimental models of organ fibrosis.^7^^,^^8^ For instance, zDHHC9 activity has been shown to protect mice from renal fibrosis, whereas zDHHC18 was found to promote this disease by mediating palmitoylation of HRAS.^7^^,^^8^ zDHHC3 and zDHHC7 have been shown to contribute to cardiac hypertrophy in mice by regulating Rac1 palmitoylation,^9^ further highlighting the potentially critical and nuanced role of the zDHHCs in fibrotic disorders.

Another member of the DHHC family, zDHHC21, has been previously shown to be involved in lipopolysaccharide- and burn-induced lung injury by regulating leukocyte infiltration and inflammation.^10^ However, the impact of zDHHC21 on lung fibroblast activation, particularly in the context of pulmonary fibrosis, has not yet been studied.

To examine the putative role of zDHHC21 in pulmonary fibrosis, we employed the zDHHC21^dep/dep^ mouse strain, characterized by the in-frame deletion of the phenylalanine in position 233 (ΔF233), resulting in a loss-of-function mutation.^11^ We found that zDHHC21^dep/dep^ mice demonstrated exacerbated lung fibrosis in response to bleomycin (Bleo) compared to wild-type (WT) animals (Figure 1A and B). Next, to evaluate ECM protein expression, we isolated lung fibroblasts (MLFs) from WT and zDHHC21^dep/dep^ mice and stimulated them with transforming growth factor-beta 1 (TGF- β1). As shown in Figure 1C, zDHHC21 loss of function enhanced the TGF-β1–induced ECM proteins expression, myofibroblast transformation compared to WT cells, suggesting that zDHHC21 suppresses pro-fibrotic signaling. Along with enhanced profibrotic phenotype in zDHHC21^dep/dep^ mice, we also observed the increased activation of PI3K/Akt/mTOR signaling pathway (Figure 1D). These findings led us to hypothesize that zDHHC21-mediated palmitoylation controls the negative regulators of the profibrotic signaling. To test this hypothesis, we first assessed the palmitoylation status of the phosphatase and tensin homolog deleted on chromosome 10 (PTEN) protein, a known modulator of the PI3K/Akt profibrotic signaling axis.^14^ Using an Acyl-Resin Assisted Capture (Acyl-RAC) assay in MLFs,^13^ we identified PTEN as a palmitoylated protein. Furthermore, TGF-β1 treatment resulted in a rapid decrease of PTEN palmitoylation, suggesting that this modification is potentially linked to PTEN’s function (Figure 1E). We also found significantly reduced palmitoylation of PTEN in MLFs isolated from zDHHC21^dep/dep^ mice (Figure 1E), indicating that zDHHC21 is the primary acyltransferase for this protein.

**Figure 1:**
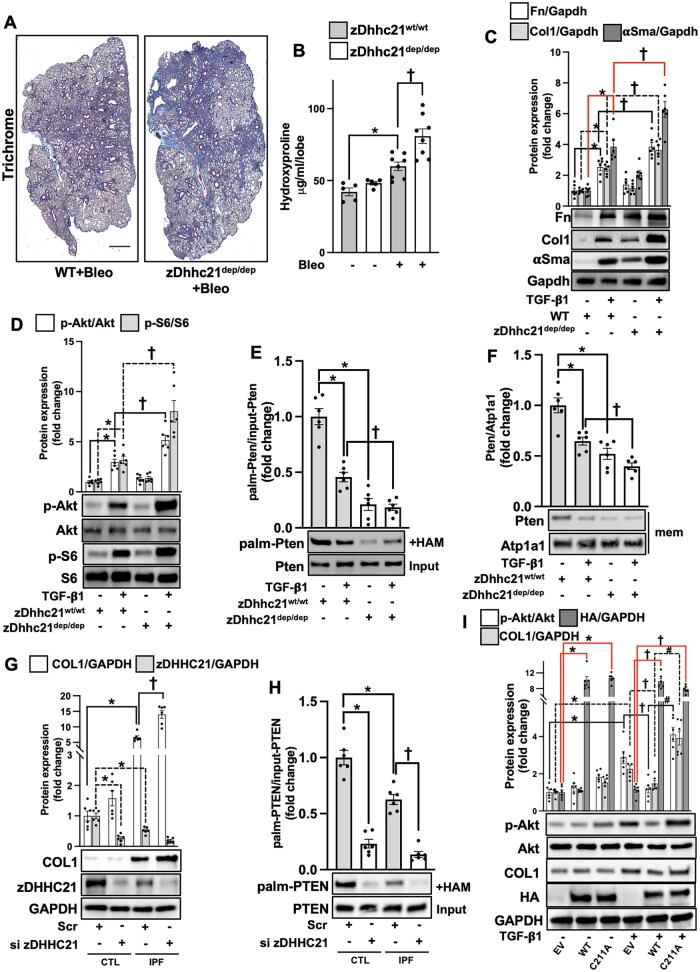
zDHHC21 loss of function increases pulmonary fibrosis and lung fibroblast activation by regulating PTEN palmitoylation. zDhhc21^wt/wt^ (WT) and zDhhc21^dep/dep^ (Dep) mice were intratracheally administered with 0.5 mg/kg of bleomycin sulfate (Bleo) to induce lung fibrosis. At day 21 after Bleo exposure, lungs were harvested, stained with Masson’s trichrome staining (A), and subjected to hyproxyproline assay (B) (*n* = 5 for sham groups, *n* = 8 for Bleo groups). (C) zDhhc21^wt/wt^ and zDhhc21^dep/dep^ MLFs were stimulated with TGF-β1 for 48 hours. Then, cells were lysed and subjected to western blotting (WB) to determine Fn, Col1, αSma, and Gapdh expressions as described previously^12^ (*n* = 6 for each group). (D) zDhhc21^wt/wt^ and zDhhc21^dep/dep^ MLFs were stimulated with TGF-β1 for 4 hours. Then, cells were lysed and subjected to WB to determine p-akt (Ser 473), total Akt, p-S6 (Ser235/236), and total S6 expressions (*n* = 6 for each group). (E) zDhhc21^wt/wt^ and zDhhc21^dep/dep^ MLFs were stimulated with TGF-β1 for 4 hours. Then, cells were lysed and subjected to Acyl-RAC as described previously.^13^ palm-Pten, Pten protein levels were measured by WB (*n* = 6). Cell lysates which were incubated with resin beads but not treated with hydroxylamine (-HAM) were used as background controls (not shown). (F) WT and zDHHC21^dep/dep^ MLFs were stimulated with TGF-β1 for 4 hours. Then, cells were lysed and subjected to cell fractionation assay to isolate membrane/organelle proteins using commercially available kit (Cat #: 9038) from Cell Signaling Technologies according to manual’s instructions. Protein levels of Pten and Atp1a1 were measured by WB (*n* = 6 each group). (G–I) Human primary control (CTL) and IPF-derived lung fibroblasts were obtained from Lonza Bioscience (Cat. #: CC-2512 and CC-7231 respectively) and transiently transfected with Scr or sizDHHC21 using iMFectin Poly DNA Transfection Reagent (GenDepot, Cat. #: I7200-101). Twenty-four hours later, cells were harvested and COL1, GAPDH, and zDHHC21 levels were detected by Western blot (G), Acyl-RAC assay to determine the levels of palmitoylated PTEN (palm-PTEN) (H) (*n* = 6 each group). (I) CTL HLFs were transfected with EV, WT-PTEN (WT), or C211A-PTEN (C211A), which were custom-made by Origene. Inc. as described in G. Then, cells were stimulated with TGF-β1 (10 ng/mL) for 24 hours. After stimulation, cells were harvested and subjected to western blot (*n* = 6 each group). Data are mean ± SEM. *P* < .05; significant comparisons by 1-way ANOVA: *vs. unstimulated or saline, ^†^vs. TGF-β1 or Bleo, ^#^vs WT + TGF-β1.

Palmitoylation is a known process to increase a protein’s hydrophobicity, resulting in the enhanced affinity toward lipid bilayers, such as the plasma membrane (PM).^3^ PTEN’s recruitment to the inner side of the PM is the critical step to properly execute its phosphatase activity and reverse PI3K/Akt signaling.^14^ Fittingly, we observed that the PTEN expression in the membrane fraction was significantly downregulated in zDHHC21^dep/dep^ cells compared to WT (Figure 1F). This result suggests that zDHHC21-mediated palmitoylation promotes PTEN’s membrane targeting, consequently modulating PI3K activity and Akt/mTOR signaling in lung fibroblasts.

We also found that the expression of zDHHC21 is downregulated in IPF human lung fibroblasts (IPF-HLF) (Figure 1G). zDHHC21 silencing resulted in higher collagen 1 expression and decreased PTEN palmitoylation in IPF-HLFs (Figure 1G and H). Of note, we obtained primary HLFs from a commercial vendor, future studies should focus on testing the role of zDHHC21 in HLFs derived from different control and IPF subjects. We also tested whether zDHHC21 can regulate the palmitoylation of another negative regulator of TGF-β1 signaling, Smad7(13). We were unable to detect palm-Smad7 in MLFs (data are available on figshare.com).

Finally, we determined which cysteine (C) residue(s) can be palmitoylated in PTEN. Previous reports have indicated that the C2 domain of PTEN, which encompasses 186–352 amino acids, plays a critical role in its recruitment to PM.^14^^,^^15^ To this end, we were interested in C211 and C218 as possible candidates for PTEN palmitoylation sites. The mutation of C211 residue to Alanine (A) (C211A) significantly downregulated PTEN palmitoylation compared to WT or C218A mutant PTEN in HEK293T cells, suggesting that C211 is a main target for palmitoylation (data are available on figshare.com). Next, we found that C211 is critical in the activation of PI3K/Akt/mTOR signaling in TGF-β1–stimulated HLFs. As shown in Figure 1I, the transfection with WT PTEN significantly downregulated the phosphorylation of Akt and collagen 1 expression in TGF-β1–activated cells. However, the transfection with mutant PTEN in C211 amino acid had no inhibitory effect on TGF-β1–induced Akt phosphorylation in HLFs. We also observed that the phosphorylation of S6 was downregulated in the presence of WT but not C211 mutant PTEN (data not shown). These results suggest that zDHHC21 loss of function impairs PTEN palmitoylation and leads to enhanced profibrotic responses in lung fibroblasts. However, its potential role in alveolar epithelial, endothelial, and immune cells during the progression of pulmonary fibrosis is currently unknown and needs to be studied in the future.

Taken together, our findings underscore the functional relevance of palmitoylating enzymes in the progression of IPF and potentially other diseases associated with dysregulated Akt/mTOR signaling. However, further studies are required to elucidate how PTEN palmitoylation influences its structure and stability, and to define the contributions of other zDHHC family members to the pathobiology of IPF and fibroblast activation.

## Supplementary Material

aanag097_Supplementary_Data
